# Supplementary material for: Cloud BioLinux: pre-configured and on-demand bioinformatics computing for the genomics community
Source: BMC Bioinformatics. 2012 Mar 19;13:42. doi: 10.1186/1471-2105-13-42 (PMC3372431; doi:10.1186/1471-2105-13-42)
Supplement: Additional file 1 — Supplementary 1 Cloud BioLinux software documentation in the form of a mini, self-contained website. Users need to download and uncompress the .zip file, and open through a web browser the "index.html" file available on the main directory. (ZIP 1823 kb). [file 1471-2105-13-42-S1.ZIP › Cloud-BioLinux-Package-Documentation/docs/caftools.html]

Bio-Linux Software Documentation Pages

Back to search form

## caftools

|  |  |
| --- | --- |
| Name | caftools |
| Description | **caftools** is distributed in the bio-linux-assembly-converstion-tools package.  This package contains a number of scripts useful for converting between different assembly formats, including formats used with assemblies of sequence generated using new sequencing technologies.  The commands available through caftools are:  - caf\_check\_pads - fix\_quals - caf\_build\_consensus - cafcat - sdict - acedb - libsrc - caf2gap - caf2ace - phrapcons - caf\_pad - caf2phrap - cafmerge - nd\_clip - templates - caf\_depad - caf2fastq For further details of these programs and how to use them, please refer to the Userguide, linked in the remote docs section below. |
| Homepage | http://www.sanger.ac.uk/Software/formats/CAF/ |
| Remote Documentation | http://www.sanger.ac.uk/Software/formats/CAF/userguide.shtml      http://www.sanger.ac.uk/Software/formats/CAF/ |
